# Oligogenic Origin of Differences of Sex Development in Humans

**DOI:** 10.3390/ijms21051809

**Published:** 2020-03-06

**Authors:** Núria Camats, Christa E Flück, Laura Audí

**Affiliations:** 1 Growth and Development Research Group, Vall d’Hebron Research Institute (VHIR), Center for Biomedical Research on Rare Diseases (CIBERER), Instituto de Salud Carlos III, Barcelona, 08035 Catalonia, Spain; 6620lap@gmail.com; 2 Pediatric Endocrinology, Diabetology and Metabolism, Department of Pediatrics and Department of BioMedical Research, Bern University Hospital and University of Bern, CH-3010 Bern, Switzerland; christa.flueck@dbmr.unibe.ch

**Keywords:** differences of sex development, DSD, oligogenicity, oligogenic disease, 46,XY DSD, 46,XX DSD, hypospadias, high throughput sequencing techniques, HTS

## Abstract

Sex development is a very complex biological event that requires the concerted collaboration of a large network of genes in a spatial and temporal correct fashion. In the past, much has been learned about human sex development from monogenic disorders/differences of sex development (DSD), but the broad spectrum of phenotypes in numerous DSD individuals remains a conundrum. Currently, the genetic cause of less than 50% of DSD individuals has been solved and oligogenic disease has been proposed. In recent years, multiple genetic hits have been found in individuals with DSD thanks to high throughput sequencing. Our group has been searching for additional genetic hits explaining the phenotypic variability over the past years in two cohorts of patients: 46,XY DSD patients carriers of *NR5A1* variants and 46,XY DSD and 46,XX DSD with *MAMLD1* variants. In both cohorts, our results suggest that the broad phenotypes may be explained by oligogenic origin, in which multiple hits may contribute to a DSD phenotype, unique to each individual. A search for an underlying network of the identified genes also revealed that a considerable number of these genes showed interactions, suggesting that genetic variations in these genes may affect sex development in concert.

## 1. Introduction

Sex development is a very complex biological event that requires the concerted collaboration of a large network of genes in a spatial and temporal correct fashion [1]. In the past, much has been learned about human sex development from monogenic disorders/differences of sex development (DSD), but the broad spectrum of phenotypes in numerous DSD individuals remains a conundrum. Currently, the genetic cause of less than 50% of DSD individuals has been solved [2,3]. Oligogenic disease has been proposed. In fact, multiple genetic hits, which might not be deleterious by themselves, have been found in individuals with DSD [4,5,6,7,8,9,10,11,12,13,14].

Oligogenic inheritance is also currently discovered for several other disorders of the endocrine systems. For instance, in congenital hypogonadotropic hypogonadism (CHH) more than 25 causative genes are now considered to explain around 50% of the cases, and in at least 20% of cases disease-causing variants in two or more genes have been identified [15,16,17,18,19,20,21]. Similarly, in congenital hypothyroidism digenic [22,23] and polygenic [24] candidate-gene variants have been associated with the phenotypes [25].

## 2. Monogenic Inheritance in Humans with DSD

In humans, the DSD phenotypes manifesting discordances among sex chromosomes, gonadal and/or genital development are classified into three groups, namely sex chromosome DSD, 46,XX DSD or 46,XY DSD [26]. Additionally, for each of the 46,XX and 46,XY DSD groups, multiple gene and corresponding protein defects have been characterized. Animal models with the corresponding gene defects have generally been able to reproduce the human phenotype. Monogenic causes for both 46,XX and 46,XY DSD include genes regulating gonadal development, gonadal and/or adrenal steroidogenesis, genital ducts’ development, and target-cell responsiveness [2,3,27,28]. Clinical and biochemical phenotypes of DSD due to genetic defects, causing (a) a complete loss of protein function resulting in complete gonadal dysgenesis (CGD), (b) classical forms of defects of steroidogenesis (both gonadal and/or adrenal), and (c) hormone resistance, mostly relate directly to the underlying genetic defect, which is then typically monogenetic. Whenever possible, genetic family studies will confirm this type of inheritance.

Defects in genes regulating gonadal development and causing gonadal dysgenesis, either complete or partial, or gonadal sex reversal include a large groups of genes in 46,XY DSD (e.g., *ARX*, *ATRX*, *CBX2*, *DAX1*, *DHH*, *DMRT1*, *EMX2*, *ESR2*, *FGFR2*, *GATA4*, *HHAT*, *MAP3K1*, *NR5A1*, *SOX9*, *SRY*, *TSPYL1*, *WNT4*, *WT1*, *ZFPM2*, and *ZNRF3*) as well as in 46,XX DSD (e.g., *BMP15*, *FGF9*, *FOXL2*, *NR2F2*, *NR5A1*, *NUP107*, *RSPO1*, *SOX3*, *SOX9*, *SOX10*, *SRY*, and *WNT4*) [3,28]. Gene defects of steroidogenesis causing DSD include *AKR1C2*, *AKR1C4*, *CYB5A*, *CYP11A1*, *CYP11B1*, *CYP17A1*, *CYP19A1*, *CYP21A2*, *DHCR7*, *HSD3B2*, *HSD17B3*, *POR*, *SRD5A2*, and *STAR* [3,28]. Gene defects affecting gonadal ducts’ development comprise *AMH* in 46,XY and *HOXA13* in 46,XX DSD [3,28]. Finally, hormone resistance syndromes causing monogenic forms of DSD are due to defects in genes *AMHR*, *AR*, *ESR1*, and *LHCGR* [3,28].

## 3. Digenic and Combined DSDs Described by Karyotyping and Single Candidate Gene Analyses

Patients carrying a digenic or combined DSD disease have been described even before the era of high throughput sequencing (HTS), when either the phenotype suggested the involvement of more than one gene defect or, incidentally, when an abnormal sex-chromosome karyotype is combined with an another gene defect [29,30,31,32,33,34,35].

Androgen receptor gene (*AR*) defects have occasionally been described in patients with a complete or partial androgen insensitivity syndrome (CAIS or PAIS) in whom sex chromosome analysis revealed a 47,XXY karyotype corresponding to a Klinefelter syndrome [29,30,31,32]. Similarly, a patient with a clinical and molecular diagnosis of familial male-limited precocious puberty due to an activating *LHCGR* mutation was demonstrated to have a 47,XXY karyotype upon detection of abnormally increased gonadotropin levels [33].

A 46,XY female DSD patient has been reported to combine *AR* and *POR* gene mutations causing a PAIS phenotype at birth, while clinical and biochemical phenotype at adrenarche suggested typical steroid anomalies of POR deficiency [34].

An adult 46,XY female DSD patient with primary amenorrhea, gonadal dysgenesis, bilateral gonadoblastoma, and dysgerminoma was first found to carry a missense *SRY* mutation, but later developed progressive focal segmental glomerular sclerosis and kidney failure. *WT1* gene analysis revealed an intron 9 splice-site mutation and confirmed Frasier syndrome [35].

## 4. Oligogenic DSDs Described by High Throughput Sequencing (HTS)

In the past decade, high throughput sequencing (HTS) has changed the genetic approach in research and diagnostics. Whole-exome sequencing (WES) has led to the discovery of many new genes and has given insight into complex traits [36,37,38]. In addition, the HTS approach is discovering that some patients carry variants in more than one gene that may contribute to their phenotype.

Oligogenic DSD origin has been revealed either as a result of HTS directly or in a second approach, when the first candidate gene detected seemed not sufficient to explain the phenotype. Several recent studies demonstrate that many patients carry a gene variant likely responsible for the phenotype together with other variants in known or new candidate genes for DSD [4,5,6,7,8,9,10,11,12,13,14].

### 4.1. Digenic and Oligogenic Origin of DSD Revealed by HTS

In sex development, digenic inheritance has recently been suggested by WES analysis in a 46,XY DSD patient with gonadal dysgenesis (*NR5A1* and *MAP3K1* variants) [7] and in a family with a 46,XY DSD male (*NR5A1* variant) and 46,XY DSD female (*NR5A1* and *TBX2* variants) [8] (Table 1).

In 46,XY patients with hypospadias, an oligogenic origin was also suggested by several HTS studies [4,5,9,10,11,12,14] (Table 1).

Kon et al. [4] analyzed 25 causative-candidate-susceptibility genes in 62 46,XY patients with non-syndromic and non-familial hypospadias. Causative mutations were described in seven patients (11.3%), with the *AR* gene accounting for the highest proportion (4/7) followed by *SRD5A2, HSD3B2,* and *BCN2*. In addition, four of these patients carried additional variants in other genes: one hemizygous *AR* patient carried heterozygous missense substitutions in *HOXB6* and *MAMLD1* and one patient with a homozygous missense mutation in *HSD3B2* had an additional heterozygous missense variant in *SRD5A2* that is reported to retain 3% of enzyme activity. The authors concluded that non-syndromic hypospadias may result from a digenic mechanism (Table 1).

Results from an analysis using a targeted DSD gene panel (including 64 known DSD and 967 candidate genes) described a total of 13 46,XY DSD patients who had more than one curated variant in diagnostic DSD genes [5]. Eight of these patients were classified as 46,XY DSD of unknown origin and five had hypospadias. Of the eight patients with 46,XY DSD of unknown origin, five had a known variant in the *AR* in combination with other DSD gene variants. Two patients had an additional pathogenic homozygous variant in *SRD5A2* or *HSD17B3*, respectively, with the patient combining *AR* and *HSD17B3* presenting a third variant in *SOX9*. Two other *AR* variant carriers had additional variants in a testis development gene, *WDR11* and *ZFPM2*, the other one carried variants in *NR5A1* and *FGFR2*. Three individuals had a pathogenic variant in a testis development gene (*MAP3K1* or *NR5A1*) in combination with a less damaging DSD gene variant in *WT1*, *LHCGR,* or *ZFPM2*. Of the five patients with hypospadias, three were found to have a likely pathogenic variant in a testis development gene (*MAP3K1* or *ZFPM2*) in combination with a variant of unknown significance (VUS) in an additional DSD gene (*WDR11, MAP3K1,* or *GATA4*), while one patient had two homozygous pathogenic variants (in *HSD3B2* and in a CHH gene, *GNRHR*) and the last patient carried variants of unknown significance (VUS) in two genes of gonadal development (*CHD7* and *DHH*) (Table 1).

In most cases with oligogenic inheritance, at least two of the genes were predicted to be pathogenic and/or contribute to the phenotype. Robevska et al. [9] analyzed the functional characteristics of *NR5A1* gene variants and the phenotypes from 15 patients (most of them described in the study of Eggers et al. [5]). Two of these patients carried each an additional heterozygous variant in known 46,XY DSD genes, *ZFPM2* [5] and *SRD5A2*. Whether these additional gene variants are able to modulate the *NR5A1* patient phenotypes has been suggested by the authors. The patient carrying an additional *ZFPM2* variant presented a more severe phenotype than another patient with the same *NR5A1* variant (p.Arg84His) only; the heterozygous *SRD5A2* variant (p.Arg227Gln) has been described in numerous patients with biallelic *SRD5A2* mutations, but also as a monoallelic finding in patients with micropenis and/or hypospadias [9] (Table 1).

Kolesinska et al. [10] analyzed 37 DSD candidate genes and 21 CHH genes in 35 46,XY DSD patients; eight (23%) were found to carry gene variations in more than one candidate gene. To evaluate whether the DSD cohort was statistically enriched for oligogenicity, they compared the results to exome sequencing data from 247 male participants in the ‘Cohorte Lausannoise’ CoLaus control population. Variants in the 37 DSD-related genes were filtered for non-synonymous variants with a MAF <1.0%, including nonsense, splice-site (±6 base pairs) and missense variants found to be damaging in at least one of the two protein prediction programs, SIFT or MutationTaster. They found a statistical enrichment in oligogenic variants in their DSD cohort compared to CoLaus controls (23% vs. 2.5%; *p* = 0.0003). From these eight patients, five combined an *AR* gene defect with another monoallelic gene defect in either a gonadal dysgenesis gene (*SOX9*, *CBX2*, or *DMRT1*) or androgen synthesis gene (*POR* or *DHCR7*). Another patient was compound heterozygous for an androgen synthesis gene (*HSD17B3*) variant and a heterozygous variant in *CYP17A1*. Still, another patient combined two gonadal dysgenesis genes (*HOXA13* and *ARX*), and one patient combined two heterozygous gene variants in the androgen synthesis gene (*AKR1C4*) and the genital duct development gene *(AMH*) (Table 1).

In a study on *GATA4* [11], two 46,XY male DSD patients with cryptorchidism, micropenis, and hypospadias without cardiac defects were heterozygous for *GATA4* missense variants located close to the *GATA4* DNA-binding site, but both variants had wild-type functional activity in vitro. HTS analysis in these two patients revealed additional gene variants in the genes for *LHCGR* and *LRP4*, respectively, which are likely contributing to the DSD phenotype [11] (Table 1).

Furthermore, analysis of 70 Chinese patients with a 46,XY DSD phenotype (who could not be diagnosed according to the typical clinical phenotypes and routine candidate gene strategies; most patients presented with undervirilization, such as microphallus, variable degrees of hypospadias, and cryptorchidism) with a candidate gene panel (33 candidates and 47 genes involved in sexual differentiation and development) revealed that 19 out of 33 patients presented multiple (two or more) gene variants, a proportion significantly higher than the rate observed in 144 individuals from a control population [12]. The highest frequency of combined variants was detected in the genes for *AR*, *SRD5A2* and *NR5A1*. Eighty percent (8 of 10) of patients carrying an heterozygous *NR5A1* variant presented additional gene variants in *SRY*, *FGF10*, *CST9*, *AR*, *MYH6*, *EGF*, *BMP2*, *SOX3*, *HSD17B3*, or *WT1* (Table 1). The authors concluded that multiple genetic lesions in some cases suggested that DSD is not a simple monogenic disorder and that a potential digenic or oligogenic pattern may underlie the pathological process [12].

In a recent study of 130 Han Chinese 46,XY males with hypospadias of variable degrees (associated or not with other DSD signs and/or other system involvement), 105 genes were analyzed on a panel including genes involved in gonadal/urogenital development (55 well recognized genes), CHH (seven genes), syndromic DSD (four genes), and others (39 genes) [14]. Genetic variants were identified in 25 patients (19.21%): 13 (52%) in the *SRD5A2* gene (compound heterozygous or homozygous), six (24%) in the *AR* gene (hemizygous), and six were heterozygous in other genes. Among them, gene variants in two different genes were only detected in three patients (12%): one compound heterozygous for *SRD5A2* and one hemizygous for *AR* carried the same *PROKR2* heterozygous mutation (p.W178S, previously reported in other Chinese patients), while another patient hemizygous for the *AR* gene carried a novel heterozygous *TRIM17* mutation (Table 1). The authors concluded that polygenic inheritance in their population may be a rare genetic cause of hypospadias. In fact, this study detected a high proportion of monogenic 46,XY DSD causes (*SRD5A2* and *AR* genes, with several mutations previously detected in the Han Chinese population), while no gene variants were detected in other candidate genes frequently found in other 46,XY DSD series such as *NR5A1* and *MAMLD1* [5,39,40].

### 4.2. HTS in a Second Approach to Detect Digenic or Oligogenic Origin of DSD

In 2012, we studied heterozygous *NR5A1* gene variants detected in 10 DSD patients (nine 46,XY DSD and one 46,XX with primary ovarian insufficiency) in whom other DSD-causing genes such as *AR*, *SRD5A2,* and *CYP17A1* were ruled out by Sanger sequencing [39]. While these gene variants were pathogenic in functional assays when tested alone, they acted similarly to wild-type when tested in heterozygote state together with the wild-type [39]. Thus, our study, similar to others [41,42,43,44], was not able to demonstrate a disease-causing effect leaving genotype–phenotype correlation for *NR5A1* variants unsolved. Many patients with variants in *NR5A1* have been described with broad phenotypes ranging from severe 46,XY DSD to unvirilized males with/without adrenal failure, 46,XX with ovarian insufficiency, 46,XX with ovotesticular DSD, and healthy carriers [45]. On the other hand, it has also been shown that SF-1 has an extraordinary network of regulators, modulators, and target genes [46,47,48].

**Figure 1:**
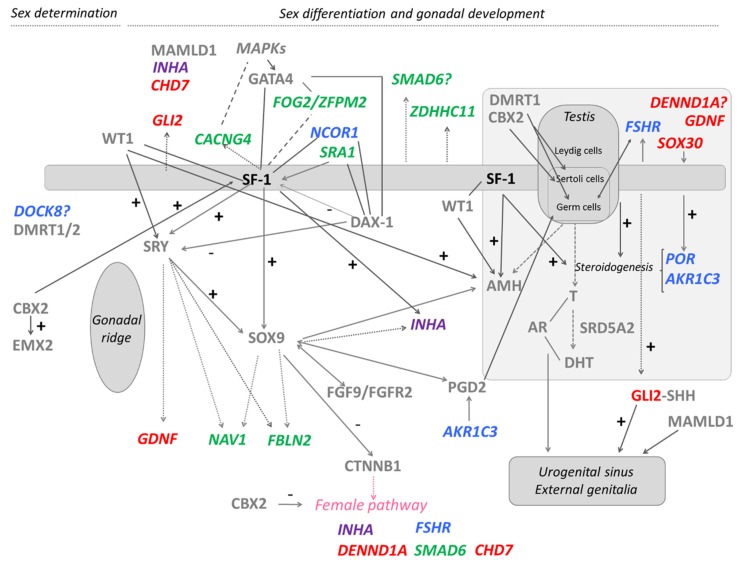
Additional genetic variants identified in four 46,XY patients with disordered/different sex development harboring heterozygous *NR5A1*/SF-1 disease-causing variants depicted with respect to the known pathways of male sex determination and differentiation. The scheme shows an overview of involved genes and their interrelationship. It emphasizes on SF-1, which seems to play an important role throughout all developmental processes (indicated by a thick line). Genes with variants identified by whole exome sequencing in the patients have specific colors. In violet: candidate gene in patient 1; in blue: candidate genes in patient 2; in green: candidate genes in patient 3; in red: candidate genes in patient 4; in grey: known genes involved in sexual development. Interrogation mark (?): function/timing/location is not clear; arrows: regulation/co-activation; dotted arrows: genes with binding regions for SF-1, SRY, and/or SOX9; lines: interaction/partnership; dashed lines: related genes, but thus far unclear how exactly; thick dashed arrows: hormone production. Modified from [13].

We therefore performed a WES analysis in four of these 46,XY DSD patients with heterozygous *NR5A1* gene defects [13] in search for additional genetic hits explaining the phenotypic variability described in patients with *NR5A1* mutations [45]. For specific bioinformatic analysis, candidate genes for DSD and genes related to *NR5A1* were collected from the literature and databases including known and potential candidate genes. Included DSD-related genes were associated to DSD conditions in humans or rodent models or related to gonadal or sex development; and SF-1/*NR5A1*-related genes were associated to SF-1 regulation or modulation. An algorithm for data analysis based on these selected project-specific DSD- and SF-1/*NR5A1*-related genes led us to identify 19 potentially deleterious variants, one to seven per patient in 18 genes (e.g., *AKR1C2*, *CACNG4*, *CHD7*, *DENND1A*, *DOCK8*, *FBLN2*, *FOG2*/*ZFPM2*, *FSHR*, *GDNF*, *GLI2*, *INHA*, *NAV1*, *NCOR1*, *POR*, *SMAD6*, *SOX30*, *SRA1*, and *ZDHHC11*) [13]. Eight of these genes had been previously reported as DSD-causing in humans. Variants related to 46,XY DSD/CHH were found in genes *CHD7*, *FOG2*/*ZFPM2*, and *SRA1*, variants related to 46,XX DSD/CHH/primary ovarian insufficiency were found in *CHD7*, *DENND1A*, *FSHR*, *GLI2*, *INHA*, *POR*, and *SRA1*. Other identified gene variants (*n* = 7) in *AKR1C3*, *DOCK8*, *NCOR1*, *FBLN2*, *NAV1*, *SMAD6*, and *GDNF* were not previously related to sex development or gonadal function. By contrast, we also observed variants in genes *CACNG4*, *ZDHHC11*, and *SOX30* that have been previously discussed as strong DSD candidates [46,49]. With respect to gene interactions with SF-1, nine of the detected genes have been previously shown to interact with SF-1/*NR5A1* in functional studies [46,47,48,50,51]: five of them (*FSHR*, *CACNG4*, *GLI2*, *SMAD6*, and *ZDHHC11*) as targets of SF-1 [46,47,48], another three (*NCOR1*, *SOX30* and *SRA1*) as regulators of SF-1 [47,50], and *INHA* as both SF-1 target and regulator [47]. From these data, we were able to construct a scheme of all hits within the landscape of currently known genes involved in male sex determination and differentiation (Figure 1). The results suggested that the broad phenotypes in heterozygous *NR5A1* 46,XY DSD subjects may be explained by an oligogenic mechanism, in which multiple hits may contribute to a DSD phenotype unique to each heterozygous SF-1/*NR5A1* individual [13] (Figure 1).

More recently, we performed a WES study in eight DSD patients (seven 46,XY and one 46,XX) carrying sequence variation in *MAMLD1* previously detected by Sanger sequencing [6]. Seven of eight *MAMLD1* sequence variations did not show alterations in functional activity in vitro when compared to wild-type MAMLD1 and thus did not explain the DSD phenotype sufficiently [40]. So far, the role of MAMLD1 in sex development is controversial for several reasons, including that the same *MAMLD1* variant may be present in healthy carriers and in 46,XY DSD patients with different severity of phenotypes [40]. We, therefore, hypothesized that *MAMLD1* variants may also not suffice to explain the 46,XY DSD phenotype. WES data from these patients were obtained and filtered by an algorithm including disease-tailored lists of *MAMLD1*-related and DSD-related genes [6]. This analysis showed that patients harbored 1–16 variants in 1–16 genes together with their *MAMLD1* variation. Fifty-five potentially deleterious variants in 41 genes were identified. Seventeen variants were reported in genes that had been previously associated with hypospadias (*ATF3*, *BNC2*, *CYP1A1*, *EMX2*, *EYA1*, *FLNA*, *GLI3*, *GRID1*, *GLI2*, *BNC2*, *FGF10*, *HOXA13*, *HSD3B2*, *IRX5*, *IRX6*, *PPARGC1B*, and *WDR11*), eight with cryptorchidism (*BNC2*, *FLNA*, *RET*, *RECQL4*, *NRP1*, *PTPN11*, *RIPK4*, and *ZBTB16*), and five with micropenis (*ZBTB16*, *BNC2*, *EVC*, *FGF10*, and *RIPK4*). Moreover, 16 identified genes had been previously described in other types of DSD (*CUL4B*, *EMX2*, *FRAS1*, *FREM2*, *HSD3B2*, *NOTCH2*, and *NRP1*) and/or were reported in different syndromes (*CYP1A1*, *EVC*, *FRAS1*, *HOXA13*, *PTPN11*, *RECQL4*, *RET*, *RIPK4*, and *ZBTB16*). In addition, 27 genes had been previously described in the context of sex or gonadal development (*ATF3*, *BNC2*, *CDH23*, *COL9A3*, *DAPK1*, *EMX2*, *EVC*, *EYA1*, *FLNA*, *FRAS1*, *FREM2*, *GLI2*, *GLI3*, *HOXA13*, *IGFBP2*, *IRX5*, *MAML3*, *MYO7A*, *NOTCH1*, *NOTCH2*, *NRP1*, *PIK3R3*, *RET*, *RIPK4*, *TGFBI*, *WNT9A*, and *WNT9B*). By contrast, only 13 genes (*ATF3*, *DAPK1*, *EMX2*, *FLNA*, *FRAS1*, *FREM2*, *GLI3*, *IGFBP2*, *IRX5*, *MAML3*, *PIK3R3*, *WNT9A*, and *WNT9B*) had been previously described in female gonadal development and 46,XX DSD and eight of them (*DAPK1*, *EMX2*, *FREM2*, *IGFBP2*, *MAML3*, *PIK3R3*, *WNT9A*, and *WNT9B*) were found in the 46,XX DSD patient (Table 1).

Nineteen of the 41 genes had been previously published in DSD panels (*ATF3*, *BNC2*, *CUL4B*, *EVC*, *FLNA*, *FRAS1*, *FREM2*, *GLI3*, *HOXA13*, *HSD3B2*, *IRX5*, *NOTCH2*, *PROP1*, *PTPN11*, *RECQL4*, *RET*, *RIPK4*, *WDR11*, *ZBTB16*). In addition, with this study we are adding 22 new candidate genes to the list of genes to consider in DSD (*CDH23*, *COL9A3*, *CYP1A1*, *DAPK1*, *EMX2*, *EYA1*, *FGF10*, *GLI2*, *GRID1*, *IGFBP2*, *IRX6*, *MAML1*, *MAML2*, *MAML3*, *MYO7A*, *NOTCH1*, *NRP1*, *PIK3R3*, *PPARGC1B*, *TGFBI*, *WNT9A*, and *WNT9B*).

**Figure 2:**
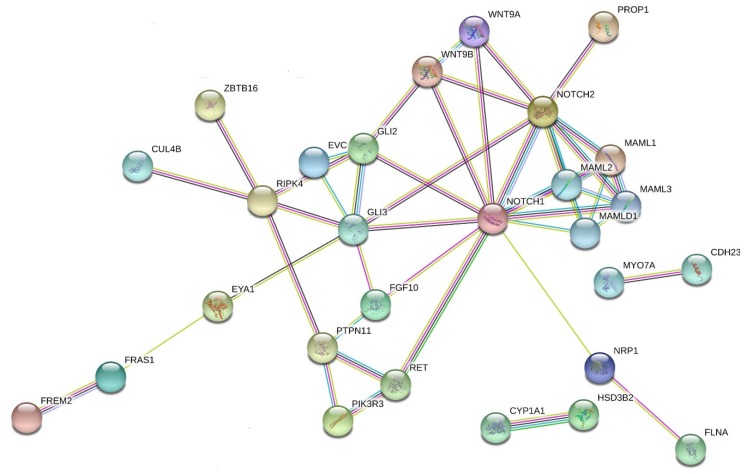
Interaction network of DSD- and *MAMLD1*-related genes identified in DSD individuals harboring genetic variants in *MAMLD1*. The scheme depicts an overview of detected genes and their interrelationship. For the search for functional human partners, we used the Search Tool for the Retrieval of Interacting Genes/Proteins (STRING, http://string-db.org/). Nodes represent proteins. Filled nodes show proteins with known or predicted 3D structure. Empty nodes depict proteins with unknown 3D structure. Candidate genes are underlined. Known interactions correspond to curated databases (turquoise lines) and experimentally determined interactions (pink lines). Predicted interactions correspond to gene neighborhood (green lines), gene fusions (red lines), and gene co-occurrence (blue lines). Other interactions correspond to text mining (yellow lines), co-expression (black lines), and protein homology (violet lines). Reproduced from [6].

A search for an underlying network comprising variants in the identified genes related to *MAMLD1* revealed a considerable number of genes (*n* = 23) that showed interactions suggesting that genetic variations in these genes may affect sex development in concert. Interestingly, *MAML3* (one variant in our 46,XX DSD patient) was found in a network related to female gonadal development [52]. Overall, three genes seemed prominent in the network analysis: *NOTCH1*/*NOTCH2* and *GLI3* (Figure 2) [6].

To summarize, results from the above studies have identified a digenic or oligogenic disorder in DSD mainly by combining mutations in genes involved more frequently in gonadal development (*NR5A1*, *MAP3K1*, *ZFPM2*, *GATA4*, *CHD7*, *HOXA13*, and *MAMLD1* combined with other gonadal development genes), followed by the *AR* gene combined with gonadal development genes and less frequently with steroidogenesis genes.

## 5. Perspectives and Pitfalls

With the rapid evolution of HTS technology in terms of improving quality and diminishing costs, it is now conceivable that the best recommendable genetic diagnostic approach in 46,XY DSD patients and 46,XX DSD non-CAH patients is using a HTS technology [3]. In this perspective, although panels of candidate genes offer guaranteed coverage, candidate gene lists are growing fast and harnessing gene panels (even economically) is getting more and more difficult. Consequently, whereas gene panels become regularly outdated, WES allows the analysis of as many genes as wanted and affording re-analyses at any demand.

On the other hand, identification of “large” CNVs has been historically restricted to other (molecular) techniques such as array-CGH. This is also being solved nowadays in gene panels, as their robust coverage allows the detection of large deletions and duplications thanks to newly developed bioinformatic tools such as ExomeDepth [53]. Furthermore, some studies demonstrate that this analysis is already possible for WES, yet it needs some improvement [54].

Nevertheless, with the identification of more variants it becomes even more difficult to distinguish between what is contributing to the phenotype and what is not. This leads to discuss the difficulties of studying polygenic disorders. In this perspective, polygenic outcome has to face the following points:

(a) Algorithms of analysis including gene lists may enrich potentially deleterious variants that may ultimately not affect sex development, yet makes the analysis easier as the gene lists can be regularly updated. On the other hand, another possibility of analysis could be a “blind” analysis following the filtering criteria, yet without filtering by the gene lists. This would give the whole picture, but many other potentially affected genes indeed without any relationship with sex development or gonadal function may arise, making the outcome harder to understand. Maybe these two approaches could be done in parallel, to have a whole picture of the patient, mainly when the gene-list approach is not giving a “clue”.

(b) Bioinformatic network analysis can help in interpreting complex genetic data and put identified single candidate genes into a greater perspective to understand their possible role in DSD biology. There are some tools, such as STRING (https://string-db.org/), BioGRID (https://thebiogrid.org/), and databases that can help, yet we have to be cautious when using these networks, as they need expert eyes to interpret them.

(c) Biostatistics requires large sample sizes mostly not available in rare disorders such as DSD. Related to this, we would like to emphasize the need for multidisciplinary teams of medical specialists and researchers to implement multicenter collaborations by using well-accredited international registries such as the I-DSD Registry [55] and the European Reference Network for Rare Endocrine Diseases (EndoERN) [56].

(d) Cell and animal models used in monogenic diseases are useful until a certain point: it is still feasible to study some gene variants and a discreet number of genes at the same time in the same model, but not in the case of “many” variants. Due to the complexity of sex development itself, a proper cell model has been searched for, for many years, to properly mimic patients’ phenotypes and study DSD-related candidate genes and role of candidate variants. Nowadays, new techniques based on cell reprograming and in vitro guided differentiation (from differentiated cells to pluripotent stem cells to redifferentiated cells again) open the field to adequate models for DSD in the near future to predict the role of candidate variants [57,58,59].

In summary, recent studies support the concept that the broad range of some DSD phenotypes may be due to digenic or oligogenic origin similarly to results described in other endocrine disorders such as CHH and congenital hypothyroidism. This is conceivable as sex developmental biology is complex and involves a vast network of genes. While some DSD phenotypes can be explained sufficiently by monogenetic defects, others may be caused by multiple minor hits in genetic networks. Whether these genes form clusters remains unknown until results of bigger cohorts of DSD individuals are studied.

## Figures and Tables

**Table 1:** Oligogenic disorders/differences of sex development (DSDs) described by high throughput sequencing (HTS) techniques.

First Gene	Other Genes	Zygosity	Variant Classification	Patient’s Phenotype	References	Method
*NR5A1*	*MAP3K1*	het/het	LP*/B	46,XY DSD GD	Mazen et al., 2016 [7]	WES
*NR5A1*	*TBX2*	het/het	P/P	46,XY DSD complete GD	Werner et al., 2017 [8]	WES
*AR*	*HOXB6, MAMLD1*	hemi/het/hemi	P/P/LB*	46,XY DSD	Kon et al., 2015 [4]	Gene panel
*HSD3B2*	*SRD5A2*	homo/het	P/P	46,XY DSD	Kon et al., 2015 [4]	Gene panel
*AR*	*SRD5A2*	hemi/homo	P/P	46,XY DSD	Eggers et al., 2016 [5]	Gene panel
*AR*	*HSD17B3, SOX9*	hemi/homo/het	P/P/VUS	46,XY DSD	Eggers et al., 2016 [5]	Gene panel
*AR*	*NR5A1, FGFR2*	hemi/het/het	P/LP/VUS	46,XY DSD	Eggers et al., 2016 [5]	Gene panel
*AR*	*ZFPM2*	hemi/het	LP/P	46,XY DSD	Eggers et al., 2016 [5]	Gene panel
*AR*	*WDR11*	hemi/het	P/VUS	46,XY DSD	Eggers et al., 2016 [5]	Gene panel
*MAP3K1*	*WT1*	het/het	LP/VUS	46,XY DSD	Eggers et al., 2016 [5]	Gene panel
*MAP3K1*	*WDR11*	het/het	LP/VUS	46,XY DSD	Eggers et al., 2016 [5]	Gene panel
*MAP3K1*	*LHCGR*	het/homo	VUS/VUS	46,XY DSD	Eggers et al., 2016 [5]	Gene panel
*ZFPM2*	*MAP3K1*	2xhet/het	LP/LP/VUS	46,XY DSD	Eggers et al., 2016 [5]	Gene panel
*NR5A1*	*ZFPM2*	het/het	P/VUS	46,XY DSD	Eggers et al., 2016 [5]; Robevska et al., 2018 [9]	Gene panel
*ZFPM2*	*GATA4*	het/het	LP/VUS	46,XY DSD	Eggers et al., 2016 [5]	Gene panel
*HSD3B2*	*GNRHR*	homo/homo	P/P	46,XY DSD	Eggers et al., 2016 [5]	Gene panel
*CHD7*	*DHH*	het/het	VUS/VUS	46,XY DSD	Eggers et al., 2016 [5]	Gene panel
*NR5A1*	*SRD5A2*	het/het	P/P	46,XY DSD	Robevska et al., 2018 [9]	Gene panel
*AR*	*SOX9, POR*	hemi/het/het	P/VUS/VUS	46,XY DSD	Kolesinska et al., 2018 [10]	Gene panel
*AR*	*CBX2*	hemi/het	VUS/VUS	46,XY DSD	Kolesinska et al., 2018 [10]	Gene panel
*AR*	*DMRT1*	hemi/het	VUS/VUS	46,XY DSD	Kolesinska et al., 2018 [10]	Gene panel
*AR*	*POR*	hemi/het	P/VUS	46,XY DSD	Kolesinska et al., 2018 [10]	Gene panel
*AR*	*DHCR7*	hemi/het	P/VUS	46,XY DSD	Kolesinska et al., 2018 [10]	Gene panel
*HSD17B3*	*CYP17A1*	comp het/het	P/P/VUS	46,XY DSD	Kolesinska et al., 2018 [10]	Gene panel
*HOXA13*	*ARX*	het/hemi	LP/VUS	46,XY DSD	Kolesinska et al., 2018 [10]	Gene panel
*AKR1C4*	*AMH*	het/het	VUS/VUS	46,XY DSD	Kolesinska et al., 2018 [10]	Gene panel
*GATA4*	*LHCGR*	het/het	VUS*/P	46,XY DSD	Martinez de LaPiscina et al., 2018 [11]	Gene panel
*GATA4*	*LRP4*	het/het	VUS*/VUS	46,XY DSD	Martinez de LaPiscina et al., 2018 [11]	Gene panel
*NR5A1*	*SRY, FGF10*	hemi/het	P/LP/VUS*	46,XY DSD	Wang H et al., 2018 [12]	Gene panel
*NR5A1*	*CST9*	het/het	LP/VUS*	46,XY DSD	Wang H et al., 2018 [12]	Gene panel
*NR5A1*	*CST9*	het/het	LP/LP*	46,XY DSD	Wang H et al., 2018 [12]	Gene panel
*NR5A1*	*AR, MYH6*	het/hemi/het	LP/LP/VUS	46,XY DSD	Wang H et al., 2018 [12]	Gene panel
*NR5A1*	*EGF*	het/het	LP/VUS	46,XY DSD	Wang H et al., 2018 [12]	Gene panel
*NR5A1*	*BMP2*	het/het	LP/LB	46,XY DSD	Wang H et al., 2018 [12]	Gene panel
*NR5A1*	*SOX3*	het/hemi	LP/LP	46,XY DSD	Wang H et al., 2018 [12]	Gene panel
*NR5A1*	*HSD17B3, WT1*	het/het/het	LP/VUS*/LP	46,XY DSD	Wang H et al., 2018 [12]	Gene panel
*SRD5A2*	*PROKR2*	comp het/het	LP/P/LP	46,XY DSD	Zhang W et al., 2019 [14]	Gene panel
*AR*	*PROKR2*	hemi/het	LP/LP	46,XY DSD	Zhang W et al., 2019 [14]	Gene panel
*AR*	*TRIM17*	hemi/het	LP/LP	46,XY DSD	Zhang W et al., 2019 [14]	Gene panel
*NR5A1*	*INHA*	het/het	P*/VUS*	46,XY DSD	Camats et al., 2018 [13]	WES
*NR5A1*	*AKR1C3*, *DOCK8*, *FSHR*, *NCOR1*, *POR*	all het, *NCOR1*: 2xhet	P*/VUS*/VUS*/VUS*/VUS*/VUS*/VUS*	46,XY DSD	Camats et al., 2018 [13]	WES
*NR5A1*	*CACNG4*, *FBLN2*, *NAV1*, *SMAD6*, *SRA1*, *ZDHHC11*, *ZFPM2*	all het	LP*/VUS*/VUS*/VUS*/LP*/LB*/VUS*/VUS*	46,XY DSD	Camats et al., 2018 [13]	WES
*NR5A1*	*CHD7*, *DENND1A*, *GDNF*, *GLI2*, *SOX30*	all het	P*/VUS*/VUS*/LB*/B*/VUS*	46,XY DSD	Camats et al., 2018 [13]	WES
*MAMLD1*	*CYP1A1, EVC, GRID1, NOTCH1, RET, RIPK4, ZBTB16*	all het, *EVC*: 2xhet, *RIPK4*: 2xhet	B*/VUS/VUS/VUS/VUS/LB/LP/VUS/VUS/VUS	46,XY DSD	Flück et al., 2019 [6]	WES
*MAMLD1*	*RECQL4*	het/het	B*/VUS	46,XY DSD	Flück et al., 2019 [6]	WES
*MAMLD1*	*GLI2, RECQL4*	het/het/het	LB*/VUS/LB	46,XY DSD	Flück et al., 2019 [6]	WES
*MAMLD1*	*CDH23, COL9A3, MAML1, NOTCH1*	all het	LB*/P/VUS/VUS/LB	46,XY DSD	Flück et al., 2019 [6]	WES
*MAMLD1*	*BNC2, FGF10, HSD3B2, IRX5, MAML2, NOTCH2*	all het	LB*/VUS/VUS/LP/VUS/VUS/VUS	46,XY DSD	Flück et al., 2019 [6]	WES
*MAMLD1*	*ATF3, BNC2, CYP1A1, EYA1, FLNA, FRAS1, GLI3, HOXA13, IRX5, IRX6, MAML1, MAML3, NRP1, PROP1, PTPN11, WDR11*	all het, *FLNA*: hemi	LB*/VUS/VUS/VUS/VUS/LB/VUS/VUS/VUS/VUS/VUS/VUS/VUS/VUS/VUS/VUS/VUS	46,XY DSD	Flück et al., 2019 [6]	WES
*MAMLD1*	*EVC, MAML3, NOTCH2, PPARGC1B, WDR11*	all het	LB*/VUS/VUS/VUS/VUS/VUS	46,XY DSD	Flück et al., 2019 [6]	WES
*MAMLD1*	*CUL4B, DAPK1, EMX2, FREM2, IGFBP2, MAML2, MAML3, MYO7A, NOTCH1, PIK3R3, TGFBI, WNT9A, WNT9B*	all het, *CUL4B*: hemi	LB/LB/VUS/VUS/LP/VUS/VUS/VUS/-/LP*/VUS/VUS/VUS/VUS/VUS	46,XX POF	Flück et al., 2019 [6]	WES

* variant classification revised in varsome/ACMG and HGMD (July 2019). GD: gonadal dysgenesis; homo: homozygous; het: heterozygous; hemi: hemizygous; B: benign; LB: likely benign; VUS: variant of uncertain significance; LP: likely pathogenic; P: pathogenic; WES: whole-exome sequencing.
